# Supplementary material for: Validation of a Real-Time PCR Assay for Identification of Fresh and Processed Carica papaya Botanical Material: Using Synthetic DNA to Supplement Specificity Evaluation
Source: Foods. 2023 Jan 25;12(3):530. doi: 10.3390/foods12030530 (PMC9913946; doi:10.3390/foods12030530)
Supplement: Supplementary file 1 [file foods-12-00530-s001.zip › foods-2145300-supplementary.pdf]

Supplementary Information: Validation of a real-time PCR assay for identification of fresh and processed *Carica papaya* botanical material: Using synthetic DNA to supplement specificity evaluation

A

Name - MK914407.1 gBlocks® Gene Fragments 269 base pairs

5'- GCG AAA TGC GAT ACT TGG TGT GAA TTG CAG AAT CCC GCG AAT CAT CGA GTC TTT GAA CGC AAG TTG CGC
CCC AGG CCT TCC GGC TGA GGG CAC GTC TGC CTG GGT GTC ACG CAC AGT CGC CCC AAA CAA AAC ATC CTT CCC
CGC GAA GGG TGT TGC TGG CTG CGG GCG GAT GCT GGC CTC CCG CCC ACG CGC CAC CCG GCC GTA GCG GTT GGC
CCA AAT ACG AAG CTC GGG CGG CGA GCG GCG CGG ACG AGT TGG TGG TTG AAC GAA CG -3'

B

Name - JX092060.1 gBlocks® Gene Fragments 239 base pairs

5'- GCG AAA TGC GAT ACT TGG TGT GAA TTG CAG AAT CCC GCG AAT CAT CGA GTC TTT GAA CGC AAG TTG CGC
CCC AGG CCA TCC GGC TGA GGG CAC GTC TGC CTG GGT GTC AGC ACA GTC GCC CCA AAA AAG ACC TCC TTC CCA
AGT CTC CCC AAG GGG GGG TGG CCT CCC GCC CGC ACG GCC GCA GCG GTT GGC CCA AAT ACG AAG CTC GGG CGG
CGA GCA GCG AGG ACG AGT TGG TGG TT -3'

Supplementary Figure S1. (A) Sequence of *Jacaratia digitata* gBlock®. (B) Sequence of *Jacaratia dolichaula* gBlock®.

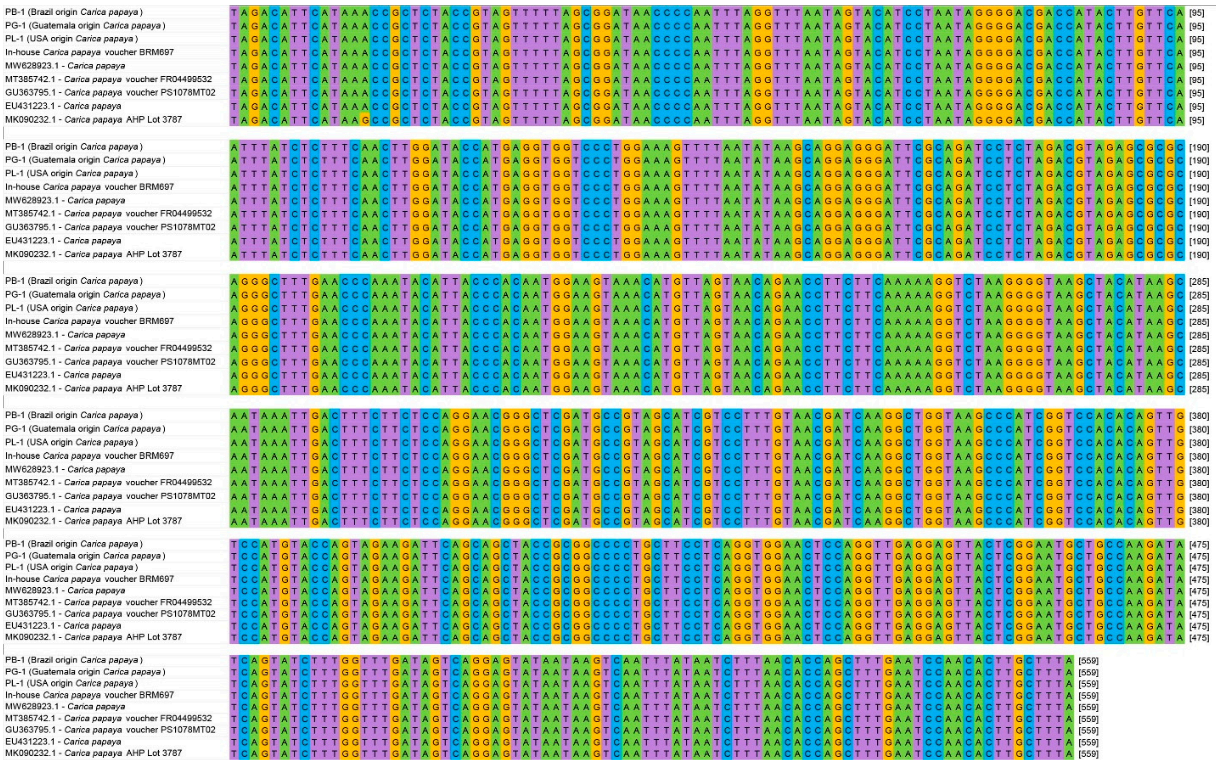

Supplementary Figure S2. Alignment of *rbcL* sequences from fresh fruit papaya samples used in this study to voucher and reference material sequences collected in house and from public databases.

Supplementary Information: Validation of a real-time PCR assay for identification of fresh and processed *Carica papaya* botanical material: Using synthetic DNA to supplement specificity evaluation

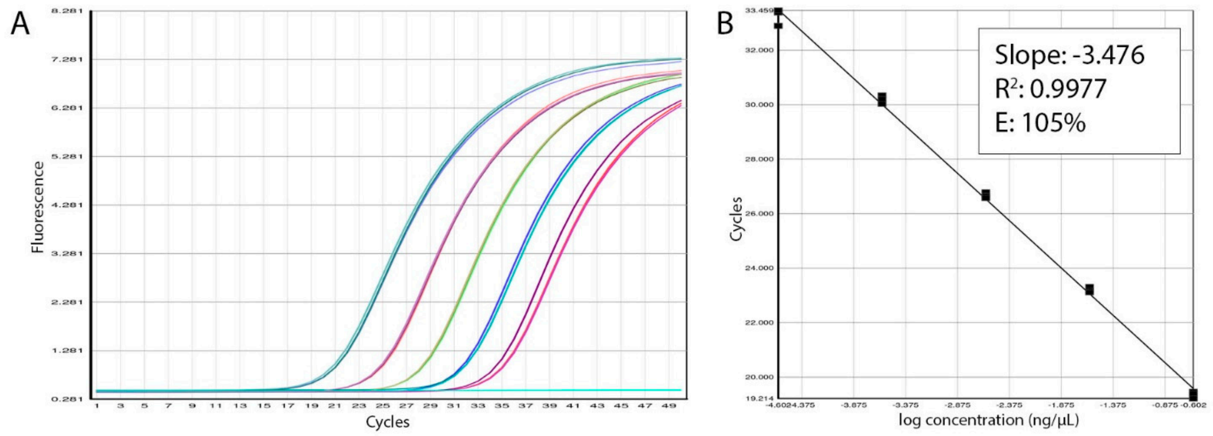

Supplementary Figure S3. (A) Standard curve created using serial dilutions of DNA extracted from USA-sourced *Carica papaya*. (B) Linearity and efficiency calculations from standard curve.

Supplementary Table S1. Repeatability evaluation using DNA from fresh *C. papaya* samples.

Sample	Target Species	Replicate	Date of analysis	Analyst	Ct	Ct Mean
**PL1 (USA)**	*Carica papaya*	1	12/09/21	RP	19.3	19.4
		2	12/09/21	RP	19.5	
		3	12/09/21	RP	19.4	
**PB-1 (Brazil)**	*Carica papaya*	1	12/09/21	RP	19.5	19.6
		2	12/09/21	RP	19.7	
		3	12/09/21	RP	19.7	
**PG-1 (Guatemala)**	*Carica papaya*	1	12/09/21	RP	20.2	20.2
		2	12/09/21	RP	20.2	
		3	12/09/21	RP	20.1	
**PL1 (USA)**	*Carica papaya*	1	12/10/21	RP	19.2	19.3
		2	12/10/21	RP	19.3	
		3	12/10/21	RP	19.3	
**PB-1 (Brazil)**	*Carica papaya*	1	12/10/21	RP	19.9	20.4
		2	12/10/21	RP	20.0	
		3	12/10/21	RP	21.2	
**PG-1 (Guatemala)**	*Carica papaya*	1	12/10/21	RP	19.4	19.5
		2	12/10/21	RP	19.5	
		3	12/10/21	RP	19.5	

Supplementary Information: Validation of a real-time PCR assay for identification of fresh and processed *Carica papaya* botanical material: Using synthetic DNA to supplement specificity evaluation

Supplementary Table S2. Reproducibility evaluation using DNA from processed *C. papaya* samples.

Sample	Target Species	Replicate	Date of analysis	Analyst	Ct	Ct Mean
**R12418 (Lot# 1036836)**	*Carica papaya*	1	12/08/21	RP	39.3	
		2	12/08/21	RP	39.4	39.5
		3	12/08/21	RP	39.8	
**R12418 (Lot# 1036836)**	*Carica papaya*	1	12/17/21	ZL	32.2	
		2	12/17/21	ZL	32.1	32.17
		3	12/17/21	ZL	32.2	
**R00003 (Lot# 1047426)**	*Carica papaya*	1	12/08/21	RP	35.8	
		2	12/08/21	RP	36.5	36.17
		3	12/08/21	RP	36.2	
**R00003 (Lot# 1047426)**	*Carica papaya*	1	12/17/21	ZL	33.0	
		2	12/17/21	ZL	33.0	32.97
		3	12/17/21	ZL	32.9	
**R00003 (Lot# 1047427)**	*Carica papaya*	1	12/08/21	RP	33.8	
		2	12/08/21	RP	33.7	33.67
		3	12/08/21	RP	33.5	
**R00003 (Lot# 1047427)**	*Carica papaya*	1	12/17/21	ZL	33.5	
		2	12/17/21	ZL	34.1	33.7
		3	12/17/21	ZL	33.5	
